# Supplementary material for: Asymptomatic immunodeficiency-associated vaccine-derived poliovirus infections in two UK children
Source: Nat Commun. 2023 Jun 9;14:3413. doi: 10.1038/s41467-023-39094-0 (PMC10251316; doi:10.1038/s41467-023-39094-0)
Supplement: Supplementary file 1 — Supplementary Information [file 41467_2023_39094_MOESM1_ESM.pdf]

Supplementary Information

A

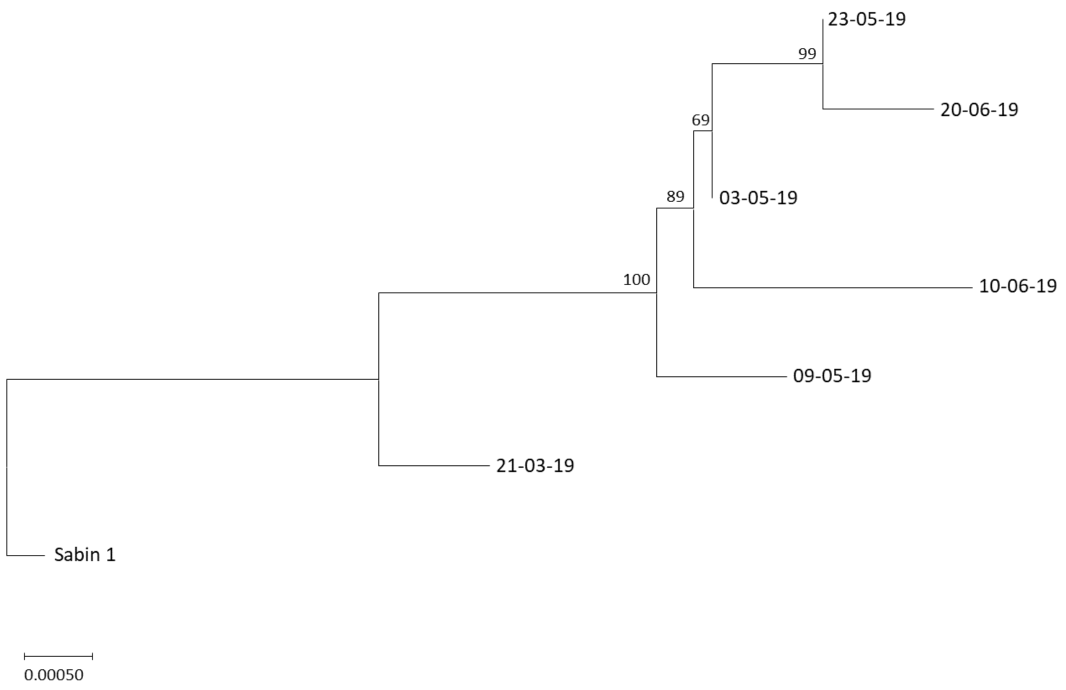

B

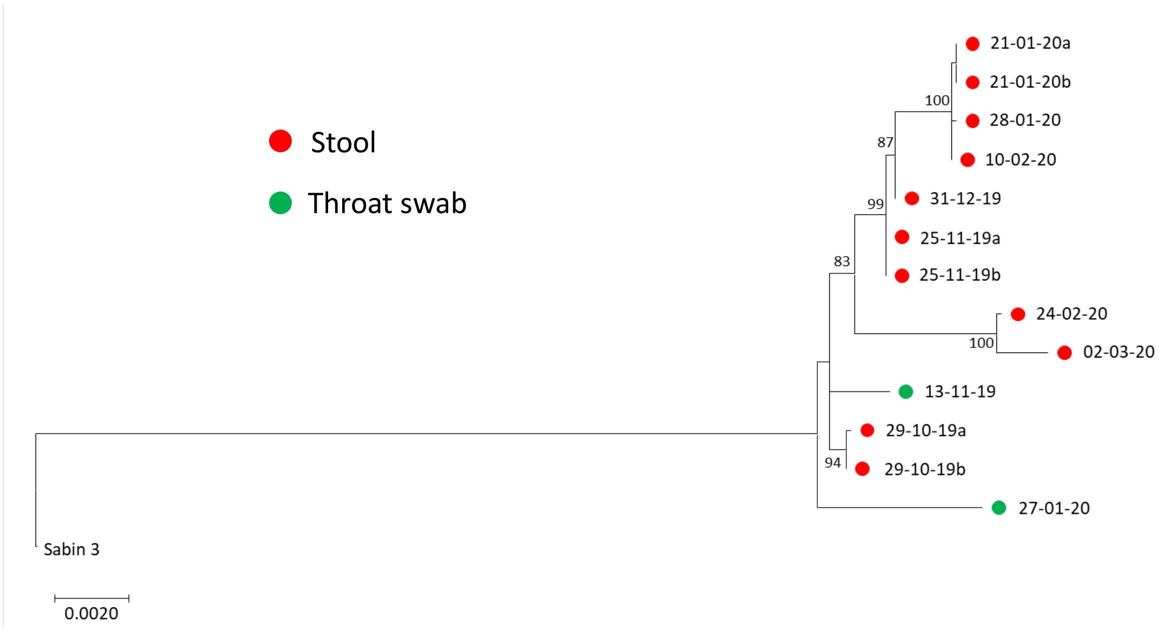

Supplementary Figure 1. Phylogenetic analysis of (A) iVDPV1 and (B) iVDPV3 strains. (A) iVDPV1 excretion was detected for 3 months since start of sampling. Whole genome sequences of 6 iVDPV1 isolates were determined by next generation sequencing. (B) iVDPV 3 excretion was detected for 4 months since start of sampling. Whole genome sequences of 13 iVDPV3 isolates (11 from stool, shown in red, and 2 from throat, shown in green) were determined by next generation sequencing.

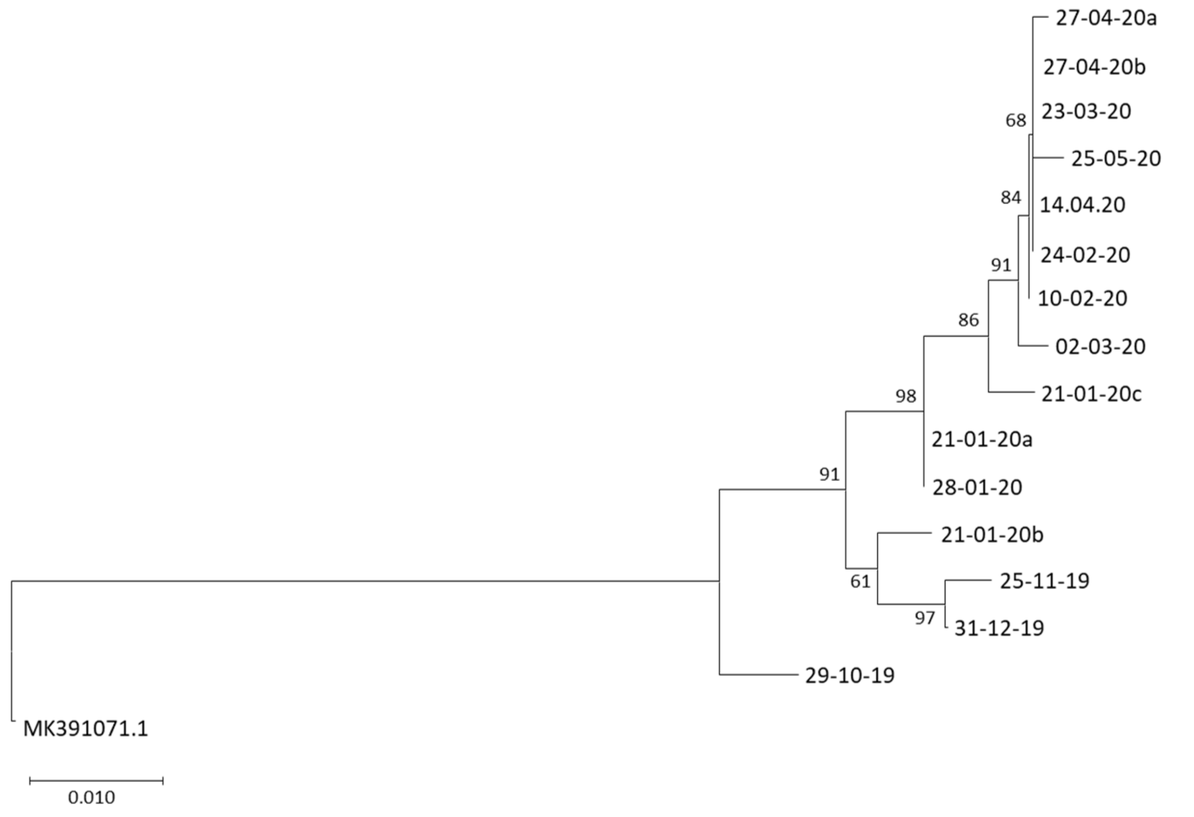

Supplementary Figure 2. Phylogenetic analysis of coxsackie A4 (CV-A4) isolates. CV-A4 excretion was detected for 7 months since start of sampling. Whole genome sequences of one CV-A4 isolate was determined by next generation sequencing. VP1 sequences from 15 CV-A4 isolates were analysed by Sangar sequencing.

Nt. Position	Change	Protein	CDS Codon No.	Codon Change	Amino Acid Change	% of isolates
318	T -> C					83%
392	G -> A					17%
436	A -> G					83%
525	T -> C					100%
808	T -> C	VP4	22	GGU -> GGC		83%
989	C -> A	VP2	14	CUG -> AUG	L -> M	67%
1012	C -> T	VP2	21	UCC -> UCT		100%
1075	G -> A	VP2	42	CUG -> CUA		100%
1204	A -> G	VP2	85	GCA -> GCG		17%
1234	T -> C	VP2	95	AAU -> AAC		17%
1264	G -> T	VP2	105	GGG -> GGT		17%
1427	A -> G	VP2	160	ACG -> GCG	T -> A	100%
1648	A -> G	VP2	233	CCA -> CCG		17%
1671	G -> A	VP2	241	AGU -> AAU	S -> N	17%
1699	C -> T	VP2	250	ACC -> ACT		17%
1714	T -> C	VP2	255	CCU -> CCC		17%
1720	C -> T	VP2	257	UGC -> UGT		17%
1732	T -> C	VP2	261	AAU -> AAC		17%
1861	A -> C	VP3	32	CCA -> CCC		17%
1939	T -> A	VP3	58	AGU -> AGA	S -> R	100%
1945	A -> T	VP3	60	AAA -> AAT	K -> N	100%
1987	C -> T	VP3	74	GAC -> GAT		100%
2075	CUA -> TUG	VP3	104	CUA -> TUG		83%
2077	A -> G	VP3	104	CUA -> CUG		17%
2278	T -> C	VP3	171	AUU -> AUC		100%
2502	G -> A	VP1	8	AGC -> AAC	S -> N	100%
2536	G -> A	VP1	19	GGG -> GGA		50%
2555	G -> C	VP1	26	GCU -> CCU	A -> P	17%
2632	C -> T	VP1	51	GCC -> GCT		83%
2641	A -> T	VP1	54	CCA -> CCT		100%
2645	G -> A	VP1	56	GUC -> AUC	V -> I	83%
2671	A -> G	VP1	64	AGA -> AGG		83%
2747	A -> T	VP1	90	AUA -> TUA	I -> L	100%
2752	C -> T	VP1	91	ACC -> ACT		17%
2766	C -> T	VP1	96	GCU -> GTU	A -> V	100%
2778	A -> G	VP1	100	AAU -> AGU	N -> S	83%
2815	T -> C	VP1	112	UAU -> UAC		100%
2926	T -> C	VP1	149	CAU -> CAC		100%
2932	A -> G	VP1	151	UUA -> UUG		100%
3001	A -> G	VP1	174	ACA -> ACG		100%
3142	G -> T	VP1	221	UCG -> UCT		17%
3436	C -> T	2A	17	UGC -> UGT		100%
3560	G -> A	2A	59	GCA -> ACA	A -> T	100%
3724	C -> T	2A	113	CUC -> CUT		100%
4116	C -> T	2B	95	ACC -> ATC	T -> I	83%
4642	G -> A	2C	173	GUG -> GUA		67%
4654	C -> T	2C	177	GAC -> GAT		17%
4864	C -> T	2C	247	GAC -> GAT		33%
4933	G -> A	2C	270	AAG -> AAA		17%
5092	T -> C	2C	323	UGU -> UGC		83%
5101	T -> C	2C	326	GCU -> GCC		83%
5110	A -> G	2C	329	CAA -> CAG		17%
5113	A -> G	3A	1	GGA -> GGG		17%
5245	C -> T	3A	45	AAC -> AAT		17%
5368	C -> T	3A	86	CAC -> CAT		100%
5458	A -> G	3C	7	GCA -> GCG		17%
5551	A -> G	3C	38	CCA -> CCG		17%
5590	C -> T	3C	51	GGC -> GGT		33%
5941	C -> T	3C	168	CAC -> CAT		83%
5953	G -> A	3C	172	GCG -> GCA		17%
5980	G -> A	3C	181	CAG -> CAA		17%
5993	A -> G	3D	3	AUC -> GUC	I -> V	17%
6009	CT -> TC	3D	8	CCU -> CTC	P -> L	33%
6070	T -> C	3D	28	AGU -> AGC		17%
6166	C -> T	3D	60	UCC -> UCT		17%
6203	C -> T	3D	73	CAC -> TAC	H -> Y	83%
6220	A -> G	3D	78	GUA -> GUG		17%
6256	C -> T	3D	90	AUC -> AUT		17%
6260	A -> G	3D	92	ACA -> GCA	T -> A	33%
6263	G -> A	3D	93	GAA -> AAA	E -> K	17%
6376	C -> T	3D	130	AUC -> AUT		83%
6391	C -> T	3D	135	ACC -> ACT		17%
6418	G -> A	3D	144	CUG -> CUA		83%
6446	C -> T	3D	154	CUG -> TUG		33%
6583	C -> T	3D	199	CAC -> CAT		17%
6922	C -> T	3D	312	ACC -> ACT		83%
7135	A -> G	3D	383	AAA -> AAG		17%
7180	T -> C	3D	398	CAU -> CAC		17%

Supplementary Figure 3. Nucleotide, codon and amino acid changes identified in iVDPV1 isolates from Child 1.

Nt. Position	Change	Protein	CDS Codon No.	Codon Change	Amino Acid Change	Variant Frequency
60	T -> C					9%
69	A -> G					9%
115	A -> T					100%
153	G -> A					100%
172	A -> T					15%
192	G -> A					100%
220	T -> A					100%
279	C -> T					100%
335	G -> T					15%
366	C -> T					100%
384	C -> T					100%
395	G -> A					100%
472	T -> C					100%
498	C -> T					100%
506	C -> T					8%
657	T -> C					8%
669	T -> C					100%
677	C -> T					100%
687	A -> G					100%
705	T -> A					100%
796	A -> T	VP4	18	CGA -> CGT		4%
847	T -> C	VP4	35	GAU -> GAC		8%
904	C -> T	VP4	54	ACC -> ACT		15%
916	G -> A	VP4	58	AAG -> AAA		100%
925	C -> T	VP4	61	CUC -> CUT		31%
946	C -> T	VP4	68	CUC -> CUT		8%
1006	C -> T	VP2	19	GGC -> GGT		100%
1012	C -> T	VP2	21	UCC -> UCT		8%
1027	G -> A	VP2	26	CAG -> CAA		15%
1042	A -> G	VP2	31	UCA -> UCG		8%
1051	T -> C	VP2	34	GCU -> GCC		54%
1071	T -> A	VP2	41	UUU -> UAU	F -> Y	100%
1084	C -> T	VP2	45	GAC -> GAT		100%
1138	C -> T	VP2	63	UUC -> UUT		8%
1156	A -> G	VP2	69	GUA -> GUG		96%
1204	A -> G	VP2	85	GCA -> GCG		100%
1210	A -> G	VP2	87	AGA -> AGG		39%
1270	T -> A	VP2	107	ACU -> ACA		100%
1276	C -> T	VP2	109	CAC -> CAT		100%
1315	C -> T	VP2	122	CUC -> CUT		100%
1354	C -> T	VP2	135	GAC -> GAT		8%
1357	T -> C	VP2	136	AGU -> AGC		100%
1360	C -> T	VP2	137	GAC -> GAT		8%
1362	A -> C	VP2	138	AAG -> ACG	K -> T	100%
1381	T -> C	VP2	144	UAU -> UAC		100%
1387	T -> G	VP2	146	AAU -> AAG	N -> K	100%
1404	GA -> AG	VP2	152	AGA -> AAG	R -> K	23%
1404	G -> A	VP2	152	AGA -> AAA	R -> K	77%
1425	A -> T	VP2	159	CAA -> CTA	Q -> L	85%
1425	AA -> TG	VP2	159	CAA -> CTG	Q -> L	15%
1440	A -> G	VP2	164	AAC -> AGC	N -> S	100%
1442	G -> A	VP2	165	GCA -> ACA	A -> T	100%
1446	T -> C	VP2	166	GUA -> GCA	V -> A	92%
1451	T -> C	VP2	168	UCC -> CCC	S -> P	8%
1480	T -> C	VP2	177	GAU -> GAC		100%
1504	A -> G	VP2	185	UUA -> UUG		4%
1522	A -> G	VP2	191	GUA -> GUG		100%
1531	T -> C	VP2	194	CAU -> CAC		100%
1535	A -> G	VP2	196	AUC -> GUC	I -> V	100%
1558	C -> T	VP2	203	AAC -> AAT		100%
1567	T -> C	VP2	206	ACU -> ACC		85%
1591	T -> C	VP2	214	GCU -> GCC		100%
1675	A -> G	VP2	242	UCA -> UCG		8%
1711	A -> G	VP2	254	CCA -> CCG		15%
1755	A -> G	VP2	269	AAA -> AGA	K -> R	100%
1774	G -> A	VP3	4	GUG -> GUA		4%
1789	T -> C	VP3	9	GGU -> GGC		8%
1804	G -> A	VP3	14	CUG -> CUA		54%
1807	G -> A	VP3	15	ACG -> ACA		100%
1849	T -> C	VP3	29	GAU -> GAC		100%
1855	T -> C	VP3	31	ACU -> ACC		23%
1861	T -> C	VP3	33	CCU -> CCC		4%

1921	T -> C	VP3	53	AUU -> AUC		8%
1938	G -> A	VP3	59	AGC -> AAC	S -> N	100%
1947	G -> A	VP3	62	AGA -> AAA	R -> K	8%
1951	C -> T	VP3	63	AAC -> AAT		100%
1972	T -> C	VP3	70	GUU -> GUC		31%
1998	C -> T	VP3	79	UCG -> UTG	S -> L	15%
2020	A -> G	VP3	86	UCA -> UCG		100%
2032	A -> T	VP3	90	GCA -> GCT		100%
2034	T -> C	VP3	91	UUU -> UCU	F -> S	100%
2041	G -> A	VP3	93	CCG -> CCA		100%
2047	G -> A	VP3	95	UUG -> UUA		100%
2128	T -> C	VP3	122	GGU -> GGC		8%
2239	C -> T	VP3	159	GGC -> GGT		31%
2285	A -> G	VP3	175	ACA -> GCA	T -> A	100%
2323	C -> T	VP3	187	GGC -> GGT		31%
2383	T -> C	VP3	207	AGU -> AGC		100%
2440	A -> T	VP3	226	CGA -> CGT		100%
2455	T -> C	VP3	231	AUU -> AUC		100%
2464	T -> C	VP3	234	UCU -> UCC		8%
2493	C -> T	VP1	6	ACU -> ATU	T -> I	100%
2547	G -> C	VP1	24	AGC -> ACC	S -> T	4%
2572	C -> T	VP1	32	GGC -> GGT		100%
2577	C -> T	VP1	34	GCG -> GTG	A -> V	8%
2602	C -> T	VP1	42	CUC -> CUT		15%
2636	G -> A	VP1	54	GCA -> ACA	A -> T	100%
2668	A -> G	VP1	64	GUA -> GUG		8%
2677	A -> G	VP1	67	CGA -> CGG		100%
2695	C -> T	VP1	73	UCC -> UCT		100%
2719	C -> A	VP1	81	CGC -> CGA		8%
2722	G -> A	VP1	82	GGG -> GGA		100%
2749	C -> T	VP1	91	GAC -> GAT		92%
2878	C -> T	VP1	134	UUC -> UUT		8%
2887	C -> T	VP1	137	ACC -> ACT		54%
2903	G -> A	VP1	143	GCU -> ACU	A -> T	100%
2908	T -> C	VP1	144	AAU -> AAC		8%
2911	T -> C	VP1	145	AAU -> AAC		100%
2977	A -> G	VP1	167	UCA -> UCG		8%
3061	A -> G	VP1	195	CCA -> CCG		54%
3079	T -> C	VP1	201	AAU -> AAC		100%
3106	C -> T	VP1	210	UUC -> UUT		8%
3163	C -> T	VP1	229	AGC -> AGT		100%
3226	T -> C	VP1	250	ACU -> ACC		8%
3232	A -> G	VP1	252	GUA -> GUG		100%
3235	C -> T	VP1	253	ACC -> ACT		12%
3247	C -> T	VP1	257	CGC -> CGT		100%
3248	A -> G	VP1	258	AUU -> GUU	I -> V	100%
3274	T -> C	VP1	266	CGU -> CGC		15%
3292	G -> A	VP1	272	CCG -> CCA		100%
3298	C -> T	VP1	274	CGC -> CGT		100%
3300	C -> T	VP1	275	GCG -> GTG	A -> V	100%
3333	G -> A	VP1	286	AGG -> AAG	R -> K	100%
3337	C -> T	VP1	287	AAC -> AAT		100%
3338	A -> G	VP1	288	AAC -> GAC	N -> D	15%
3373	A -> T	VP1	299	ACA -> ACT		15%
3376	T -> C	VP1	300	UAU -> UAC		100%
3391	G -> A	2A	5	CAG -> CAA		23%
3421	G -> A	2A	15	AAG -> AAA		31%
3446	A -> G	2A	24	AAG -> GAG	K -> E	100%
3470	A -> T	2A	32	AGC -> TGC	S -> C	100%
3508	A -> G	2A	44	UCA -> UCG		8%
3523	C -> T	2A	49	ACC -> ACT		39%
3538	G -> A	2A	54	AGG -> AGA		8%
3550	T -> A	2A	58	AAU -> AAA	N -> K	69%
3589	C -> T	2A	71	UAC -> UAT		69%
3590	C -> T	2A	72	CCU -> TCU	P -> S	15%
3607	A -> G	2A	77	GGA -> GGG		15%
3628	G -> A	2A	84	GAG -> GAA		8%
3721	T -> C	2A	115	UGU -> UGC		100%
3724	A -> G	2A	116	CAA -> CAG		15%
3742	C -> T	2A	122	AUC -> AUT		8%
3754	T -> A	2A	126	GGU -> GGA		8%
3835	C -> T	2B	4	AAC -> AAT		100%
3838	T -> C	2B	5	UAU -> UAC		100%

3841	T -> C	2B	6	AUU -> AUC		100%
3844	G -> A	2B	7	GAG -> GAA		100%
3847	A -> T	2B	8	UCA -> UCT		15%
3862	C -> T	2B	13	UUC -> UUT		4%
3928	G -> T	2B	35	ACG -> ACT		100%
3931	T -> C	2B	36	AUU -> AUC		100%
3937	G -> A	2B	38	GAG -> GAA		92%
3976	G -> A	2B	51	CUG -> CUA		100%
3994	T -> C	2B	57	AAU -> AAC		8%
4003	T -> C	2B	60	GAU -> GAC		100%
4018	C -> T	2B	65	CUC -> CUT		8%
4086	CT -> TC	2B	88	ACU -> ATC	T -> I	100%
4135	A -> G	2C	7	AAA -> AAG		100%
4147	G -> A	2C	11	GCG -> GCA		100%
4186	A -> G	2C	24	AAA -> AAG		100%
4201	T -> C	2C	29	AUU -> AUC		4%
4208	T -> C	2C	32	UUG -> CUG		31%
4212	G -> A	2C	33	AGA -> AAA	R -> K	100%
4232	G -> A	2C	40	GCC -> ACC	A -> T	31%
4309	A -> G	2C	65	CAA -> CAG		100%
4321	T -> C	2C	69	AGU -> AGC		4%
4339	T -> C	2C	75	AUU -> AUC		100%
4381	A -> G	2C	89	AGA -> AGG		100%
4401	T -> A	2C	96	CUU -> CAU	L -> H	100%
4444	T -> C	2C	110	AAU -> AAC		100%
4495	G -> A	2C	127	GUG -> GUA		8%
4537	A -> G	2C	141	CUA -> CUG		100%
4615	C -> T	2C	167	UAC -> UAT		100%
4636	C -> T	2C	174	AUC -> AUT		69%
4672	T -> C	2C	186	GAU -> GAC		8%
4690	A -> G	2C	192	CAA -> CAG		15%
4708	G -> A	2C	198	GAG -> GAA		100%
4732	G -> A	2C	206	CUG -> CUA		100%
4748	C -> T	2C	212	CUG -> TUG		100%
4795	C -> T	2C	227	AUC -> AUT		15%
4798	A -> G	2C	228	ACA -> ACG		8%
4828	G -> T	2C	238	CUG -> CUT		100%
4855	T -> C	2C	247	GAU -> GAC		100%
4868	G -> A	2C	252	GGC -> AGC	G -> S	100%
4882	A -> G	2C	256	AGA -> AGG		15%
4975	T -> A	2C	287	GGU -> GGA		8%
5005	C -> T	2C	297	UCC -> UCT		100%
5017	C -> T	2C	301	UAC -> UAT		69%
5050	T -> C	2C	312	AAU -> AAC		8%
5068	C -> T	2C	318	UCC -> UCT		100%
5125	G -> A	3A	8	UUG -> UUA		100%
5166	A -> G	3A	22	AAU -> AGU	N -> S	15%
5170	C -> T	3A	23	GAC -> GAT		92%
5176	C -> T	3A	25	CUC -> CUT		100%
5182	A -> G	3A	27	GCA -> GCG		100%
5245	C -> T	3A	48	AGC -> AGT		100%
5275	G -> A	3A	58	AGG -> AGA		15%
5320	T -> C	3A	73	GCU -> GCC		100%
5374	T -> C	3B	4	ACU -> ACC		8%
5380	A -> G	3B	6	UUA -> UUG		100%
5389	A -> C	3B	9	AAA -> AAC	K -> N	8%
5401	G -> A	3B	13	GUG -> GUA		100%
5419	A -> T	3B	19	GCA -> GCT		8%
5419	A -> G	3B	19	GCA -> GCG		15%
5425	A -> G	3B	21	GUA -> GUG		100%
5428	A -> G	3B	22	CAA -> CAG		15%
5431	G -> A	3C	1	GGG -> GGA		31%
5470	C -> T	3C	14	AAC -> AAT		100%
5500	G -> A	3C	24	GAG -> GAA		100%
5521	C -> T	3C	31	CAC -> CAT		4%
5524	C -> T	3C	32	GAC -> GAT		100%
5566	C -> T	3C	46	AGC -> AGT		31%
5626	A -> G	3C	66	GCA -> GCG		15%
5644	C -> T	3C	72	AUC -> AUT		100%
5647	T -> C	3C	73	ACU -> ACC		100%
5737	C -> T	3C	103	AUC -> AUT		8%
5743	C -> T	3C	105	AAC -> AAT		15%
5791	A -> G	3C	121	GAA -> GAG		100%

5827	T -> A	3C	133	GCU -> GCA		15%
5854	C -> T	3C	142	ACC -> ACT		15%
5872	T -> C	3C	148	GGU -> GGC		100%
5905	G -> A	3C	159	GGG -> GGA		100%
5929	A -> G	3C	167	UCA -> UCG		100%
5950	G -> A	3C	174	CUG -> CUA		31%
6067	C -> T	3D	30	UUC -> UUT		100%
6134	A -> G	3D	53	AAC -> GAC	N -> D	100%
6148	A -> G	3D	57	GCA -> GCG		100%
6194	C -> T	3D	73	CAC -> TAC	H -> Y	100%
6220	T -> C	3D	81	UAU -> UAC		69%
6241	A -> G	3D	88	CUA -> CUG		100%
6253	A -> G	3D	92	ACA -> ACG		100%
6256	A -> G	3D	93	GAA -> GAG		54%
6298	A -> G	3D	107	CUA -> CUG		100%
6313	G -> A	3D	112	UUG -> UUA		54%
6344	A -> T	3D	123	AUG -> TUG	M -> L	8%
6406	A -> G	3D	143	AAA -> AAG		100%
6418	A -> G	3D	147	ACA -> ACG		85%
6439	G -> A	3D	154	CUG -> CUA		100%
6445	T -> C	3D	156	ACU -> ACC		8%
6448	T -> C	3D	157	UAU -> UAC		8%
6511	T -> C	3D	178	GCU -> GCC		100%
6574	C -> T	3D	199	CAC -> CAT		100%
6577	A -> G	3D	200	AAA -> AAG		100%
6580	C -> T	3D	201	AAC -> AAT		100%
6590	A -> G	3D	205	AUA -> GUA	I -> V	100%
6607	A -> G	3D	210	GUA -> GUG		100%
6640	T -> C	3D	221	AUU -> AUC		15%
6643	G -> A	3D	222	CCG -> CCA		100%
6679	C -> T	3D	234	UAC -> UAT		100%
6850	A -> G	3D	291	UCA -> UCG		100%
6925	A -> G	3D	316	AUA -> AUG	I -> M	8%
6952	C -> T	3D	325	GCC -> GCT		8%
7003	C -> T	3D	342	CUC -> CUT		8%
7062	T -> C	3D	362	AUA -> ACA	I -> T	100%
7132	C -> T	3D	385	CCC -> CCT		100%
7165	A -> G	3D	396	GAA -> GAG		8%
7192	A -> G	3D	405	AAA -> AAG		100%
7201	G -> T	3D	408	CGG -> CGT		4%
7201	G -> C	3D	408	CGG -> CGC		96%
7204	T -> C	3D	409	AAU -> AAC		100%
7207	G -> A	3D	410	ACG -> ACA		23%
7253	G -> A	3D	426	GAA -> AAA	E -> K	15%
7258	A -> G	3D	427	GAA -> GAG		100%
7298	A -> G	3D	441	AUC -> GUC	I -> V	100%
7303	A -> G	3D	442	GGA -> GGG		100%

Supplementary Figure 4. **Nucleotide, codon and amino acid changes identified in iVDPV3 isolates from Child 2.**

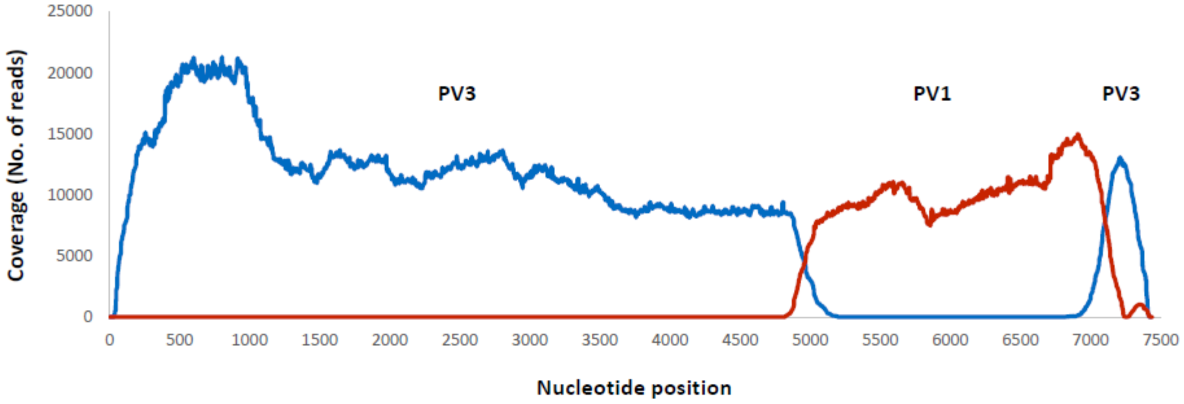

Supplementary Figure 5. Double recombinant structure of a Sabin 3/Sabin 1/Sabin 3 isolate excreted by Child 2. A double recombinant structure was shown by all iVDPV3 isolates.
