# Supplementary material for: Asymptomatic immunodeficiency-associated vaccine-derived poliovirus infections in two UK children
Source: Nat Commun. 2023 Jun 9;14:3413. doi: 10.1038/s41467-023-39094-0 (PMC10251316; doi:10.1038/s41467-023-39094-0)
Supplement: Supplementary file 3 — Reporting Summary [file 41467_2023_39094_MOESM3_ESM.pdf]

nature portfolio

Corresponding author(s): Maria Zambon and Anika Singanayagam

Last updated by author(s): May 1, 2023

Reporting Summary

Nature Portfolio wishes to improve the reproducibility of the work that we publish. This form provides structure for consistency and transparency in reporting. For further information on Nature Portfolio policies, see our Editorial Policies and the Editorial Policy Checklist.

Statistics

For all statistical analyses, confirm that the following items are present in the figure legend, table legend, main text, or Methods section.

n/a	Confirmed	
☐	☒	The exact sample size (*n*) for each experimental group/condition, given as a discrete number and unit of measurement
☐	☒	A statement on whether measurements were taken from distinct samples or whether the same sample was measured repeatedly
☐	☒	The statistical test(s) used AND whether they are one- or two-sided<br>*Only common tests should be described solely by name; describe more complex techniques in the Methods section.*
☐	☒	A description of all covariates tested
☐	☒	A description of any assumptions or corrections, such as tests of normality and adjustment for multiple comparisons
☐	☒	A full description of the statistical parameters including central tendency (e.g. means) or other basic estimates (e.g. regression coefficient) AND variation (e.g. standard deviation) or associated estimates of uncertainty (e.g. confidence intervals)
☐	☒	For null hypothesis testing, the test statistic (e.g. *F*, *t*, *r*) with confidence intervals, effect sizes, degrees of freedom and *P* value noted<br>*Give P values as exact values whenever suitable.*
☒	☐	For Bayesian analysis, information on the choice of priors and Markov chain Monte Carlo settings
☒	☐	For hierarchical and complex designs, identification of the appropriate level for tests and full reporting of outcomes
☒	☐	Estimates of effect sizes (e.g. Cohen's *d*, Pearson's *r*), indicating how they were calculated

Our web collection on statistics for biologists contains articles on many of the points above.

Software and code

Policy information about availability of computer code

Data collection	No software was used
Data analysis	Statistical analysis and molecular clock based inference of the date of OPV administration leading to the vaccine-derived PV (VDPV) were done using GraphPad Prism version 9 software (https://www.graphstats.net/). PV sequencing data were processed and analysed using Geneious 10.2.3 and, MEGA X software. Demultiplexing of Illumina sequencing was performed on MiSeq Reporter softward v2. FASTQ sequencing were adapter and quality trimmed by Cutadapt v2.10.

For manuscripts utilizing custom algorithms or software that are central to the research but not yet described in published literature, software must be made available to editors and reviewers. We strongly encourage code deposition in a community repository (e.g. GitHub). See the Nature Portfolio guidelines for submitting code & software for further information.

Data

Policy information about availability of data

All manuscripts must include a data availability statement. This statement should provide the following information, where applicable:

- Accession codes, unique identifiers, or web links for publicly available datasets
- A description of any restrictions on data availability
- For clinical datasets or third party data, please ensure that the statement adheres to our policy

Raw fastq NGS files are available from NCBI’s Sequence Read Archive under project code PRJNA924856. Nucleotide consensus sequences for poliovirus isolates are

available from GenBank with accession numbers OQ286202- OQ286220. Nucleotide consensus sequences for Coxsackievirus A4 isolates are available from GenBank with accession numbers OQ319970-OQ319985. Sabin vaccine strain reference genome sequences used had GenBank accession codes AY184219 (https://www.ncbi.nlm.nih.gov/sra/?term=AY184219) and AY184221 (https://www.ncbi.nlm.nih.gov/sra/?term=AY184221).

Human research participants

Policy information about studies involving human research participants and Sex and Gender in Research.

Reporting on sex and gender	Both of the case descriptions were of 2 year old children who were of the male sex (biologically assigned sex). Data is presented for each case individually.
Population characteristics	Both cases are male children aged 2 years old with a diagnosis of primary immunodeficiency and on treatment with intravenous immunoglobulin.
Recruitment	Both participants were recruited after they tested positive for poliovirus on stool samples in the community.
Ethics oversight	Informed consent was received from parents/caretakers and consent was obtained to publish. The processing of patient data by Public Health England (and the successor organisation UK Health Security Agency) was conducted under Regulation 3 of The Health Service (Control of Patient Information) Regulations 2002, permitting the processing of confidential patient information for communicable disease and other risks to public health. Processing and analysis of individual patient-level data by clinicians were undertaken in compliance with the Data Protection Act.

Note that full information on the approval of the study protocol must also be provided in the manuscript.

Field-specific reporting

Please select the one below that is the best fit for your research. If you are not sure, read the appropriate sections before making your selection.

☒ Life sciences ☐ Behavioural & social sciences ☐ Ecological, evolutionary & environmental sciences

For a reference copy of the document with all sections, see nature.com/documents/nr-reporting-summary-flat.pdf

Life sciences study design

All studies must disclose on these points even when the disclosure is negative.

Sample size	This is a case series with a sample size of two. No sample size calculation was performed as the sample size was not pre chosen.<br><br>Transgenic mouse assays are based on several years of development work comparing results with those known for assays in monkeys and molecular assays measuring mutations known to have an impact on neurovirulence. Numbers of animals and doses were determined as such giving clear dose responses and sufficient to differentiate viruses known to have different neurovirulence properties. An effort was made to minimise the number of animals for ethical reasons.
Data exclusions	No data was excluded.
Replication	Mouse experiments (at least 8 mice per group) were performed in duplicate. Data from both experiments are presented in the manuscript. All attempts at replication were successful.
Randomization	Randomisation is not relevant for the case series of n=2, (which is descriptive with no intervention group).<br><br>In Figure 4, mice and cages were randomised before the experiment to experimental groups.
Blinding	Blinding is not relevant for the case series of n=2, (which is descriptive with no intervention group).<br><br>In Figure 4, animals were randomly assigned to experimental groups. Samples were blinded to the NIBSC animal testing group who do not know which samples are used in an experimental groups.

Reporting for specific materials, systems and methods

We require information from authors about some types of materials, experimental systems and methods used in many studies. Here, indicate whether each material, system or method listed is relevant to your study. If you are not sure if a list item applies to your research, read the appropriate section before selecting a response.

Materials & experimental systems

n/a	Involved in the study
☒	☐ Antibodies
☐	☒ Eukaryotic cell lines
☒	☐ Palaeontology and archaeology
☐	☒ Animals and other organisms
☐	☒ Clinical data
☒	☐ Dual use research of concern

Methods

n/a	Involved in the study
☒	☐ ChIP-seq
☒	☐ Flow cytometry
☒	☐ MRI-based neuroimaging

Eukaryotic cell lines

Policy information about cell lines and Sex and Gender in Research

Cell line source(s)	Rhabdomyosarcoma (RD) cells and L20B cells are acquired via the Global Polio Laboratory Network as per the WHO Polio Laboratory Manual (WHO/IVB/04.10). L20B cells are a genetically engineered mouse cell line expressing the human poliovirus receptor. RD cells are derived from a human rhabdomyosarcoma.
Authentication	RD and L20B cells were obtained from CDC, Atlanta (USA), and are used across the WHO Global Polio Laboratory Network (GPLN) for poliovirus surveillance. Cells were authenticated by post thaw cell morphology analysis, DNA barcoding of the Cytochrome Oxidase Subunit 1(CO1) Mitochondrial Region and DNA profiling using Short Tandem Repeat (STR) profiling (RD cells only).
Mycoplasma contamination	All cell lines tested negative for mycoplasma.
Commonly misidentified lines (See ICLAC register)	No commonly misidentified cell lines were used.

Animals and other research organisms

Policy information about studies involving animals; ARRIVE guidelines recommended for reporting animal research, and Sex and Gender in Research

Laboratory animals	Tg21-Bx transgenic mice expressing the human poliovirus receptor, 50% male, 50% female, 6-8 weeks-old. Mice were housed in in compliance with the UK government Code of Practice for the Animals (Scientific Procedures) Act 1986 - https://assets.publishing.service.gov.uk/government/uploads/system/uploads/attachment_data/file/388535/CoPanimalsWeb.pdf
Wild animals	No wild animals were used
Reporting on sex	Sex was considered in the study design of animal experiments. 50% biological male and 50% biological female mice were used (assigned sex). Data disaggregated for sex was not collected.
Field-collected samples	Mice were housed in in compliance with the UK government Code of Practice for the Animals (Scientific Procedures) Act 1986 - https://assets.publishing.service.gov.uk/government/uploads/system/uploads/attachment_data/file/388535/CoPanimalsWeb.pdf
Ethics oversight	Animal work was approved by NIBSC's Ethics and Human Materials Advisory Committees. NIBSC's Animal Welfare and Ethical Review Body approved the application for Procedure Project Licence Number 70/8979 which was approved by the UK Government Home Office and under which animal care and protocols shown in this paper were conducted. All animal care and protocols used at NIBSC adhere to UK regulations (Animals, scientific procedures, Act 1986 that regulates the use of animals for research in the UK) and to European Regulations (Directive 2010/63/Eu of the European Parliament on the protection of animals used for scientific purposes).

Note that full information on the approval of the study protocol must also be provided in the manuscript.

Clinical data

Policy information about clinical studies

All manuscripts should comply with the ICMJE guidelines for publication of clinical research and a completed CONSORT checklist must be included with all submissions.

Clinical trial registration	*Provide the trial registration number from ClinicalTrials.gov or an equivalent agency.*
Study protocol	*Note where the full trial protocol can be accessed OR if not available, explain why.*
Data collection	*Describe the settings and locales of data collection, noting the time periods of recruitment and data collection.*
Outcomes	*Describe how you pre-defined primary and secondary outcome measures and how you assessed these measures.*
